# Yoda1 Inhibits TGFβ-Induced Cardiac Fibroblast Activation via a BRD4-Dependent Pathway

**DOI:** 10.3390/cells14131028

**Published:** 2025-07-04

**Authors:** Perwez Alam, Sara M. Stiens, Hunter J. Bowles, Hieu Bui, Douglas K. Bowles

**Affiliations:** 1 Department of Biomedical Sciences, University of Missouri, Columbia, MO 65211, USA; 2 Dalton Cardiovascular Research Center, University of Missouri, Columbia, MO 65211, USA

**Keywords:** cardiac fibroblast, fibroblast activation, ion channels, Yoda1, BRD4, mechanosensitive channels, Periostin

## Abstract

Fibrosis represents a pivotal pathological process in numerous diseases, characterized by excessive deposition of extracellular matrix (ECM) that disrupts normal tissue architecture and function. In the heart, cardiac fibrosis significantly impairs both structural integrity and functional capacity, contributing to the progression of heart failure. Central to this process are cardiac fibroblasts (CFs), which, upon activation, differentiate into contractile myofibroblasts, driving pathological ECM accumulation. Transforming growth factor-beta (TGFβ) is a well-established regulator of fibroblast activation; however, the precise molecular mechanisms, particularly the involvement of ion channels, remain poorly understood. Emerging evidence highlights the regulatory role of ion channels, including calcium-activated potassium (K_Ca_) channels, in fibroblast activation. This study elucidates the role of ion channels and investigates the mechanism by which Yoda1, an agonist of the mechanosensitive ion channel Piezo1, modulates TGFβ-induced fibroblast activation. Using NIH/3T3 fibroblasts, we demonstrated that TGFβ-induced activation is regulated by tetraethylammonium (TEA)-sensitive potassium channels, but not by specific K⁺ channel subtypes such as BK, SK, or IK channels. Intriguingly, Yoda1 was found to inhibit TGFβ-induced fibroblast activation through a Piezo1-independent mechanism. Transcriptomic analysis revealed that Yoda1 modulates fibroblast activation by altering gene expression pathways associated with fibrotic processes. Bromodomain-containing protein 4 (BRD4) was identified as a critical mediator of Yoda1’s effects, as pharmacological inhibition of BRD4 with JQ1 or ZL0454 suppressed TGFβ-induced expression of the fibroblast activation marker Periostin (Postn). Conversely, BRD4 overexpression attenuated the inhibitory effects of Yoda1 in both mouse and rat CFs. These results provide novel insights into the pharmacological modulation of TGFβ-induced cardiac fibroblast activation and highlight promising therapeutic targets for the treatment of fibrosis-related cardiac pathologies.

## 1. Introduction

Fibrosis is a key pathological process in many diseases, where the activation of fibroblasts leads to the excessive deposition of extracellular matrix (ECM), resulting in tissue scarring. In the heart, this process significantly contributes to conditions like heart failure, disrupting the heart’s normal structure and function. Cardiac fibroblasts (CFs) are the third most abundant cell type in the myocardium and play a crucial role in maintaining the structural integrity of the heart, especially after injury [1,2]. Cardiac stress due to damage or inflammation activates quiescent CFs as one of the primary responders to stress. Activated fibroblasts proliferate and, in chronic conditions, transform into myofibroblasts. These activated myofibroblasts help protect the injured tissue in the short term by promoting healing. However, in chronic injury or pathological conditions, myofibroblasts contribute to ECM deposition and also acquire contractile function by upregulating the expression of contractile proteins such as alpha-smooth muscle actin (α-SMA) and non-muscle myosin, thus becoming the main effector cell type for fibrosis [3].

A central player in this process is transforming growth factor-beta (TGFβ), which serves as a master regulator of fibroblast activation. TGFβ triggers the proliferation, migration, and differentiation of fibroblasts into myofibroblasts, setting the stage for ECM deposition. Excessive cardiac ECM can block vital survival signals for cardiomyocytes and trigger inflammation, potentially leading to cell death [4,5]. These changes can cause arrhythmias by disrupting electrical conduction [6,7]. Altered ECM structure affects heart contractile function, with atrial fibrosis being linked to cardiomyopathy and heart failure [8,9]. TGFβ can activate CFs through Smad-dependent [10] or through non-canonical, Smad-independent mechanisms [11,12,13]. In addition, ion channels have emerged as critical regulators of fibroblast function during fibrosis.

Calcium-activated potassium channels (K_Ca_), particularly K_Ca_3.1, play a role in cardiac fibroblast activation [14,15,16,17,18,19]. Activation of K_Ca_3.1 increases the driving force for calcium influx, while inhibition suppresses calcium entry [18,20]. In doing so, K_Ca_3.1 influences various cellular processes, such as cell proliferation and differentiation [21,22]. Additionally, studies show that blocking K_Ca_3.1 channels with the pharmacological inhibitor TRAM-34 can reduce angiotensin II-induced cardiac fibrosis by lowering the number of fibroblast precursor cells [23].

In addition to K_Ca_ channels, mechanosensitive ion channels influence how fibroblasts respond to mechanical and chemical stress in the heart [24]. Mechanosensitive ion channels help fibroblasts detect mechanical stress and regulate ion flow, which is crucial for their activation, differentiation, and remodeling of the ECM [24]. For instance, TRPM7 helps drive TGFβ-induced arterial fibrosis by allowing calcium to enter cells, promoting fibroblast proliferation and differentiation [25]. Similarly, TRPV4 and TRPC6 are involved in fibroblast activation and ECM production, especially during stress or injury [26]. Additionally, studies have shown that Piezo1 affects TGFβ expression, influencing fibroblast activation in cardiac fibroblasts [27].

Despite the growing body of literature highlighting the role of ion channels in cardiac fibroblast activation, the specific ion channels involved in TGFβ-induced activation and underlying mechanisms remain poorly understood. In this study, we performed a series of in vitro experiments using NIH/3T3 and primary cardiac fibroblasts, along with various pharmacological compounds, to modulate these channels and evaluate their effects on fibroblast activation following TGFβ treatment. Our results reveal that Yoda1 is a potent inhibitor of TGFβ-induced activation of cardiac fibroblasts, independent of Piezo1 channels. Instead, the effect of Yoda1 is mediated through inhibition of bromodomain-containing protein 4 (BRD4), as the Yoda1 effect was mitigated by its overexpression.

## 2. Materials and Methods

### 2.1. 3T3 Cell Culture and Yoda1 Treatment

NIH/3T3 cells, a mouse fibroblast cell line, were procured from ATCC. 3T3 cells were cultured in DMEM/F-12 medium supplemented with 10% Serum Plus II (DF10) and maintained at 37 °C in a humidified atmosphere containing 5% CO_2_. For experiments, approximately 70,000 cells per well were seeded into 12-well plates, and treatments were initiated once the cells reached 60–70% confluence. Due to the high mortality of 3T3 cells in serum-deprived medium, all treatments were performed in 2% serum-supplemented medium (DF2). The study included four experimental groups: a control group, which received DF2 medium only; a TGFβ-treated group, in which DF2 was supplemented with 10 ng/mL TGFβ; a Yoda1-treated group, in which DF2 contained 10 µM Yoda1; and a combination group, in which DF2 was supplemented with both 10 µM Yoda1 and 10 ng/mL TGFβ. The Yoda1 + TGFβ group received a 20 min pre-treatment of Yoda1 alone followed by the addition of TGFβ. Each condition was tested in quadruplicate. Cells were incubated at 37 °C with 5% CO_2_ for 24 h during the treatment period before RNA isolation and subsequent analysis.

### 2.2. Treatment of 3T3 Cells with Ion Channel Modulators and Pharmacological Inhibitors

Studies on 3T3 cells with the pharmacologic agents listed below included the same four experimental conditions described in the experiments with Yoda1, with the pharmacologic agents replacing Yoda1. Groups treated with both TGFβ and a pharmacologic agent received a 20 min pre-treatment with the agent prior to the addition of TGFβ. Each condition was tested in quadruplicate. Cells were incubated at 37 °C with 5% CO_2_ for 24 h during the treatment period before RNA isolation and subsequent analysis. The working concentrations of each pharmacological compound are shown below (Table 1).

### 2.3. Murine Cardiac Fibroblast Isolation and Culture

Mouse and rat cardiac fibroblasts were isolated from adult (≥12 weeks old) animals. For CF isolation, the heart was excised from the animal, followed by perfusion and washing with sterile PBS under aseptic conditions. The heart tissues were minced into small fragments of approximately 1–2 mm^3^ and transferred into a dissociation medium composed of PBS supplemented with 600 U/mL Collagenase IV, 1.2 U/mL Dispase, 15 U/mL DNase I, and 1 mg/mL BSA, adjusted to pH 7.4. The digestion process was carried out at 37 °C with intermittent mixing and repeated until the tissues were fully dissociated. The resulting cell suspension was centrifuged at 4000–5000 RPM for 5–7 min, and the cell pellets were suspended in 10% serum-supplemented (FBS; Corning, Tewksbury, MA, USA; Catalog#35015CV) medium before being seeded into culture flasks.

Cells were allowed to adhere for three hours at 37 °C with 5% CO_2_ in the incubator, after which non-adherent cells were removed by washing with warm PBS, and fresh medium was added. The cultures were maintained in a humidified incubator at 37 °C with 5% CO_2_ for an additional 24 h, with media replacement as necessary. Upon reaching confluence, cells were designated as passage zero. For subsequent experiments, 5–7 × 10^4^ cells per well were seeded into 12-well plates.

### 2.4. Treatment of Murine Primary Cardiac Fibroblasts

For the drug treatment of murine primary cardiac fibroblasts, cells not exceeding passage 2 were seeded in 12-well plates at a density of 5–7 × 10^4^ cells per well. Once cells reached 70–80% confluency, they were serum-starved by washing the cells with warm PBS, followed by adding serum-free medium. Cells were incubated for 24 h before treatment with TGFβ and/or drug treatment as previously described. Cells were harvested for RNA isolation and subsequent analysis 24 h post-treatment.

### 2.5. RNA Isolation

Total RNA from the cells was extracted using the RNeasy Plus Mini Kit (Qiagen, Germantown, MD, USA; Catalog# 74136) according to the manufacturer’s protocol. RNA purity and concentration were determined using a NanoDrop spectrophotometer, and the extracted RNA was stored at −80 °C until further analysis. Complementary DNA (cDNA) was synthesized from total RNA extracted from 3T3 cells and mouse CFs using the SuperScript™ IV VILO™ Master Mix (Invitrogen, Carlsbad, CA, USA; catalog #11-756-050). The SuperScript™ III First-Strand Synthesis System (Invitrogen, Carlsbad, CA, USA; catalog# 18-080-051) was used to prepare the cDNA from rat RNA samples.

### 2.6. Transcriptomic Sequencing

RNA sequencing (RNA-seq) was performed using the RNA isolated from the 3T3 cells from four experimental groups: the control (no treatment), TGFβ-treated group, Yoda1-treated group, and Yoda1 + TGFβ combination group. Four samples from each group were used for sequencing.

RNA sequencing was performed by the MU DNA Core Facility (University of Missouri, Columbia, MO, USA). To construct Illumina Stranded mRNA libraries, poly-A-containing mRNA was extracted from total RNA, followed by RNA fragmentation. Double-stranded cDNA was synthesized from the fragmented RNA, and unique dual-index adapters were ligated to the fragment ends. A total of 16 libraries were prepared and sequenced together using an Illumina NovaSeq flow cell with a paired-end, 100-base-read-length strategy. The sequencing process yielded approximately one billion reads, corresponding to around 50 million read pairs per sample. Downstream analysis and visualization of the next-generation sequencing (NGS) data were conducted by the MU Informatics Research Core Facility (IRCF, University of Missouri, Columbia, MO, USA).

The RNA-Seq data were processed and analyzed using established protocols [28]. In brief, latent Illumina adapter sequences were identified and removed from the raw 100-mer RNA-Seq data using Cutadapt. Reads were subsequently trimmed and filtered to eliminate low-quality nucleotide calls and entire low-quality reads using the Fastx-Toolkit. To generate a final set of high-quality RNA-Seq reads, contaminant sequences were removed through similarity matching against the Phi-X genome (NC_001422.1), relevant ribosomal RNA genes obtained from the National Center for Biotechnology Information (NCBI), and repeat elements from RepBase using Bowtie. Following quality control, the processed RNA-Seq reads were aligned to the *Mus musculus* genome assembly GRCm39 using the Bioconductor package Rsubread, with default settings optimized for paired-end reads. The Rsubread algorithm employs a “seed-and-vote” mapping approach to efficiently align subreads for gene expression analysis. Initial gene expression estimates were assigned Entrez Gene IDs using the Bioconductor database org.Mm.eg.db. These estimates were subsequently normalized and arranged into a comparative matrix based on genotype using the Bioconductor package DESeq2.

Pairwise differential expression analyses were performed, comparing (i) control versus TGFβ, where TGFβ-treated samples were assessed relative to the control, and (ii) TGFβ versus Yoda1 + TGFβ, where the Yoda1 + TGFβ group was evaluated against the TGFβ-treated samples. The final results were compiled into an Excel file and visualized using principal component analysis (PCA) and volcano plots, highlighting genes with a log2(fold change) greater than 2 and adjusted *p*-values below 0.05. The genes with the most significant adjusted *p*-values were further analyzed using the Database for Annotation, Visualization, and Integrated Discovery (DAVID) and Ingenuity Pathway Analysis (IPA, version #145030503) software [29,30,31,32,33].

### 2.7. siRNA Transfection

To knock down Piezo1 in mouse cardiac fibroblasts, we used a specific siRNA targeting Piezo1. Scrambled siRNA served as a transfection control (siControl). Cells were transfected with 200 nM siRNA using LyoVec™ (InvivoGen, San Diego, CA, USA; Catalog # lyec-1) according to the manufacturer’s protocol. Briefly, cells were pre-treated with NATE™ (InvivoGen, Catalog # lyec-nate) at the recommended volume and incubated for over 30 min. In parallel, siRNA was mixed with LyoVec™ to form transfection complexes and incubated at room temperature for 30 min. The resulting siRNA-LyoVec™ complexes were then added directly to the cells and incubated for 24 h prior to serum starvation and subsequent experiments.

### 2.8. RT-PCR Analysis

Gene expression analysis was performed using RT-PCR using the QuantStudio™ 3 Real-Time PCR System (Applied Biosystems, Carlsbad, CA, USA) with iQ SYBR Green Supermix (Bio-Rad, Hercules, CA, USA) in a final reaction volume of 20 µL. Each reaction mixture contained 2 µL of cDNA, 2 µL of gene-specific primers, and nuclease-free water. The annealing temperature was set at 60 °C. Fluorescence data were collected during the 72 °C extension step of each cycle, as well as during the melt curve analysis. Gene expression quantification and data analysis were performed using QuantStudio™ 3 software (Design & Analysis 2.6.0). The specific mouse and rat primers used for the RT-PCR are shown below (Table 2).

### 2.9. BRD4 Overexpressing Adenoviral Vector Production and Cell Transduction

Mouse BRD4 complete cDNA was cloned into an adenoviral vector under the regulation of the CMV promoter, with a fluorescent reporter tag inserted downstream within the same construct. A CMV-GFP construct served as the control. Chimeric plasmid assembly and adenoviral packaging were performed by Vector Builder. For transduction, murine primary cardiac fibroblasts were infected with adenoviruses at a multiplicity of infection (MOI) of 500. Following a 24 h transduction, cells were serum-starved for an additional 24 h. Both BRD4-overexpressing and control groups were stratified into four treatment conditions: untreated, Yoda1-treated, TGFβ-treated, and Yoda1+TGFβ-treated. After 24 h of treatment, cells were harvested for RNA isolation and subsequent gene expression profiling.

## 3. Statistical Analysis

All data are presented as mean ± SE. One-way or two-way ANOVA was used for all group comparisons as appropriate, and significance was defined as *p* ≤ 0.05. Student’s *t*-test was applied for comparisons between the two groups. Statistical cutoffs for differentially expressed genes were −1.0 ≤ log^2^FC ≥ 1.0 change in expression with an adjusted *p* < 0.05.

## 4. Results

### 4.1. Potassium Channels Do Not Significantly Contribute to TGFβ-Induced NIH/3T3 Fibroblast Activation

**Figure 1:**
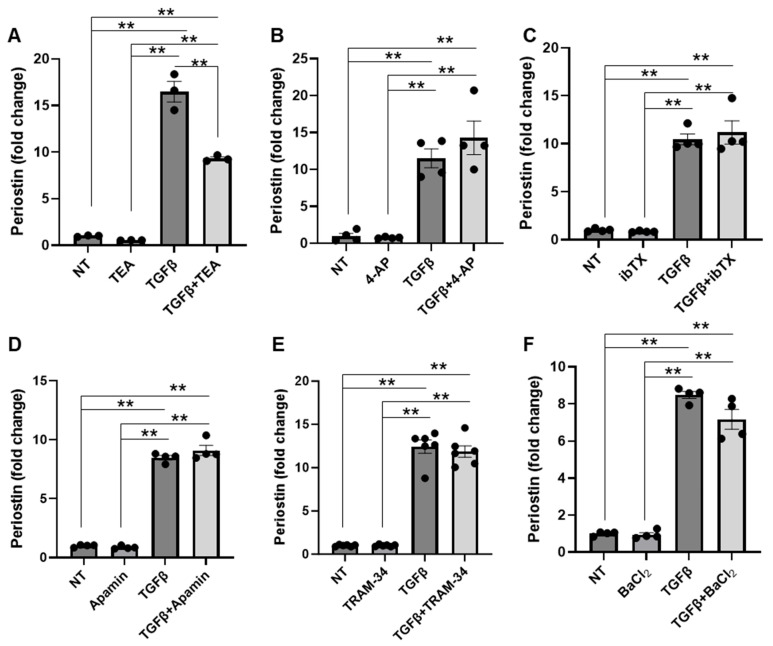
K^+^ channels do not significantly contribute to TGFβ-induced NIH/3T3 fibroblast activation. Bar graphs showing the expression of the activated fibroblast marker Periostin following treatment with (**A**) tetraethylammonium (TEA;10 mM), which inhibits both voltage-dependent and calcium-activated channels, (**B**) 4-AP (2 mM), which selectively inhibits voltage-dependent potassium (K_v_) channels, (**C**) iberiotoxin (IbTX; 100 nM), which inhibits large-conductance calcium-activated potassium channels (BK), (**D**) apamin (100 nM), which inhibits small-conductance calcium-activated potassium channels (SK), (**E**) TRAM-34 (100 nM), which inhibits intermediate-conductance calcium-activated potassium channels (IK, K_Ca_3.1), and (**F**) BaCl_2_ (10 µM), which inhibits inward-rectifying potassium channels (K_ir_). Results are presented as mean ± SE. Statistical significance between groups was assessed using one-way ANOVA. A *p*-value ≤ 0.05 was considered statistically significant; ** = *p* ≤ 0.01. NT = no treatment.

K^+^ channels regulate intracellular calcium signaling and membrane potential, both of which are essential for controlling various cellular processes involved in fibroblast activation. To assess the contribution of specific K^+^ channels in fibroblast activation, we conducted an in vitro study using NIH/3T3 fibroblasts treated with TGFβ in the presence or absence of specific potassium channel inhibitors. Fibroblast activation was assessed by analyzing the expression of the fibroblast activation marker, Periostin (Postn). Our results revealed a significant inhibition of fibroblast activation following TGFβ treatment when tetraethylammonium (TEA)-sensitive channels were inhibited (Figure 1A). However, TGFβ-induced fibroblast activation was unaffected by inhibition of voltage-dependent (K_v_) channels using 4-AP (Figure 1B), large-conductance K_Ca_ channels (BK) with iberiotoxin (IbTX; Figure 1C), small-conductance K_Ca_ (SK) channels with apamin (Figure 1D), intermediate-conductance K_Ca_ (IK, K_Ca_3.1) channels using TRAM-34 (Figure 1E), or inward-rectifying K^+^ channels (K_ir_) with barium chloride (BaCl_2_; Figure 1F). These findings suggest that a TEA-dependent mechanism contributes to TGFβ-induced fibroblast activation, while major K^+^ channels such as K_v_, BK, SK, IK, and K_ir_ are not significantly involved.

### 4.2. Yoda1 Inhibits Fibroblast Activation Through a Piezo1-Independent Mechanism

**Figure 2:**
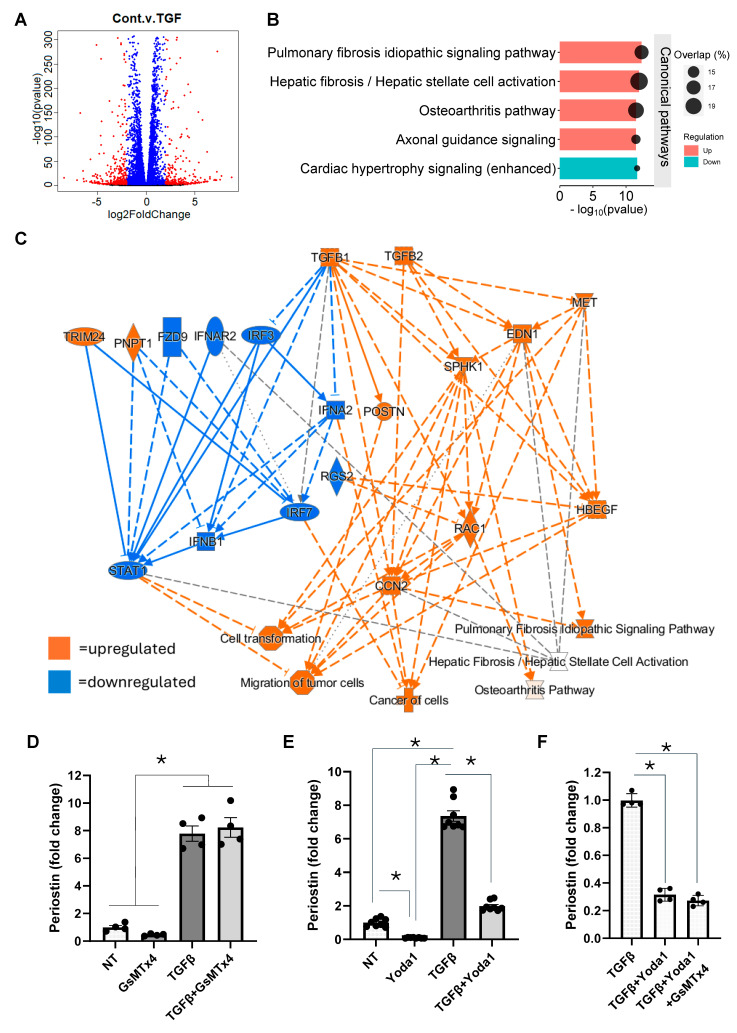
TGFβ induces a signature of fibrotic gene expression in NIH/3T3 fibroblasts, which is reversed by Yoda1 in a Piezo1-independent mechanism. (**A**) The volcano plot illustrates the genes that are significantly upregulated (orange) or downregulated (blue) in response to TGFβ. RNA sequencing revealed that 2110 genes were differentially expressed in TGFβ-treated NIH/3T3 fibroblasts compared to control, with 856 genes upregulated and 1254 genes downregulated. (**B**) Ingenuity Pathway Analysis (IPA) mapped the signature of differentially expressed genes to known signaling pathways. The five best matching pathways are displayed, where the teal blue bar indicates downregulation and the red bars represent upregulation, based on the differential gene expression in the data set. The size of the bubble corresponds to the number of overlapped genes in respective biological pathways. (**C**) IPA was used to correlate differentially expressed genes associated with cell functions and disease processes. The network illustrates the expression pattern of genes and their effects on associated pathways. Blue represents downregulation/inhibition, while orange denotes upregulation. (**D**,**E**) Bar graphs showing the effects of GsMTx4, a specific inhibitor of Piezo1, and Yoda1, a Piezo1 agonist, on the expression of Periostin with or without TGFβ treatment in NIH/3T3 fibroblasts. (**F**) Bar graph demonstrating the effect of Yoda1 alone or in combination with GsMTx4 on the TGFβ-induced Periostin expression. Results are presented as mean ± SE. Statistical significance was determined using one-way ANOVA, with *p* value ≤ 0.05 considered statistically significant. * = *p* ≤ 0.05. NT = no treatment.

To more broadly investigate the molecular mechanisms underlying TGFβ-induced fibroblast activation, RNA sequencing was performed on NIH/3T3 fibroblasts treated with or without TGFβ. The analysis revealed that 856 genes were upregulated by more than two-fold (*p* ≤ 0.05), and 1254 genes were downregulated by more than two-fold (*p* ≤ 0.05) in the TGFβ-treated samples compared to controls (Figure 2A). As anticipated, pathway analysis of the differentially expressed genes (DEGs) using Ingenuity Pathway Analysis (IPA, Qiagen) identified fibrosis-related pathways as the most significantly affected. The top pathways predicted to be positively affected by TGFβ treatment included idiopathic pulmonary fibrosis signaling and hepatic fibrosis/stellate cell activation, as well as the osteoarthritis pathway and axonal guidance signaling. In contrast, cardiac hypertrophy signaling was predicted to be downregulated in the TGFβ-treated group (Figure 2B). To link specific DEGs to potentially affected pathways following TGFβ treatment, we performed a correlation analysis using IPA. Our findings revealed significant upregulation of pathways involved in cell transformation, pulmonary and hepatic fibrosis, and tumor cell migration (Figure 2C). The prediction that TGFβ induces fibrotic signaling pathways such as those involved in idiopathic pulmonary fibrosis and hepatic fibrosis is based on upregulation of genes such as connective tissue growth factor (CCN2), a known regulator of fibrosis [34,35]; endothelin 1 (EDN1), a potent vasoconstrictor with pro-fibrotic effects [36,37]; and MET protooncogene, which is involved in cell proliferation, migration, and tissue repair [38]. A focused analysis of ion channel expression identified Piezo1 as the most highly expressed ion channel in NIH/3T3 fibroblasts (Table 3), leading us to investigate the role of Piezo1 in TGFβ-induced fibroblast activation using Piezo1-specific agonists and antagonists (Figure 2D–F). As mentioned earlier, fibroblast activation was assessed by analyzing the expression of the fibroblast activation marker, Periostin. Surprisingly, we did not observe any effect on TGFβ-induced fibroblast activation in the presence of the Piezo1-specific inhibitor, GsMTx4 (Figure 2D). Interestingly, in contrast to previous reports, treatment with the Piezo1 agonist, Yoda1, inhibited both basal and TGFβ-induced Periostin (Figure 2E). Moreover, the addition of the Piezo1 antagonist, GsMTx4, did not reverse the inhibitory effect of Yoda1 (Figure 2F).

**Figure 3:**
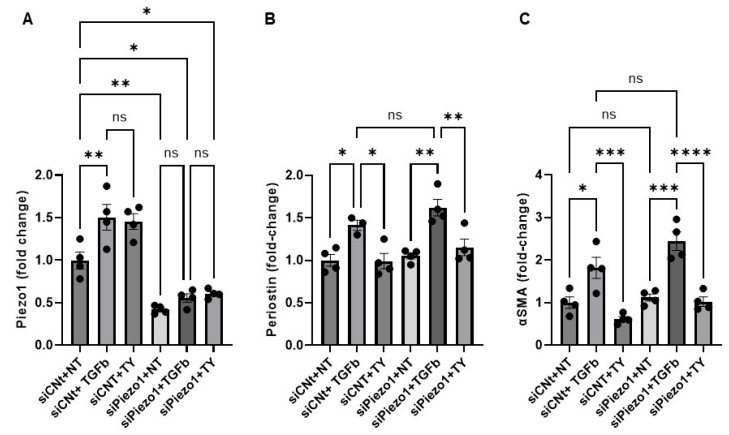
Piezo1 knockdown does not significantly affect Yoda1-mediated inhibition of TGFβ-induced fibroblast activation: (**A**) Bar graph represents the siPiezo1 (200 nM)-mediated inhibition of the Piezo1 in cardiac fibroblast. (**B**) Bar graph demonstrates the Periostin expression in the different study groups (no treatment, TGFβ, or TGFβ + Yoda1), with or without Piezo1 knockdown. (**C**) Bar graph demonstrates the αSMA expression in the different study groups (no treatment, TGFβ, or TGFβ + Yoda1), with or without Piezo1 knockdown. Results are presented as mean ± SE. Statistical significance was determined using one-way ANOVA, with *p* value ≤ 0.05 considered statistically significant. * = *p* ≤ 0.05. ** = *p* ≤ 0.01. *** = *p* ≤ 0.001. **** = *p* < 0.0001. ns = non-significant. NT = no treatment. TY = TGFβ + Yoda1.

Furthermore, to evaluate the direct role of Piezo1 in Yoda1-mediated inhibition of TGFβ-induced fibroblast activation, we employed a specific siRNA targeting Piezo1 (siPiezo1) to achieve gene knockdown in cardiac fibroblasts. Scrambled siRNA-transfected groups were considered as transfection control. TGFβ and Yoda1 treatments were carried out as described previously to induce fibroblast activation and probe the potential modulatory effects of Piezo1. Our results show that siPiezo1 treatment led to a significant reduction in Piezo1 expression compared to cells treated with scrambled siRNA (siControl) (Figure 3A). To assess the functional impact of Piezo1 knockdown, cells were divided into three treatment groups, untreated, TGFβ alone, or TGFβ combined with Yoda1, under both siPiezo1 and siControl conditions. Despite a greater than 50% knockdown of Piezo1, no significant differences were observed in the expression levels of Periostin (Figure 3B) or α-SMA (Figure 3C) in the treatment groups, no treatment, TGFβ, or TGFβ + Yoda1, when compared to their respective siControl counterparts. These findings suggest that although Piezo1 may contribute to stress-induced activation of fibroblasts, it is unlikely to be a major mediator of TGFβ-induced fibroblast activation or Yoda1-mediated suppression of TGFβ-driven activation.

### 4.3. BRD4 Identified as a Potential Mediator of Yoda1-Induced Suppression of TGFβ-Induced Fibroblast Activation

**Figure 4:**
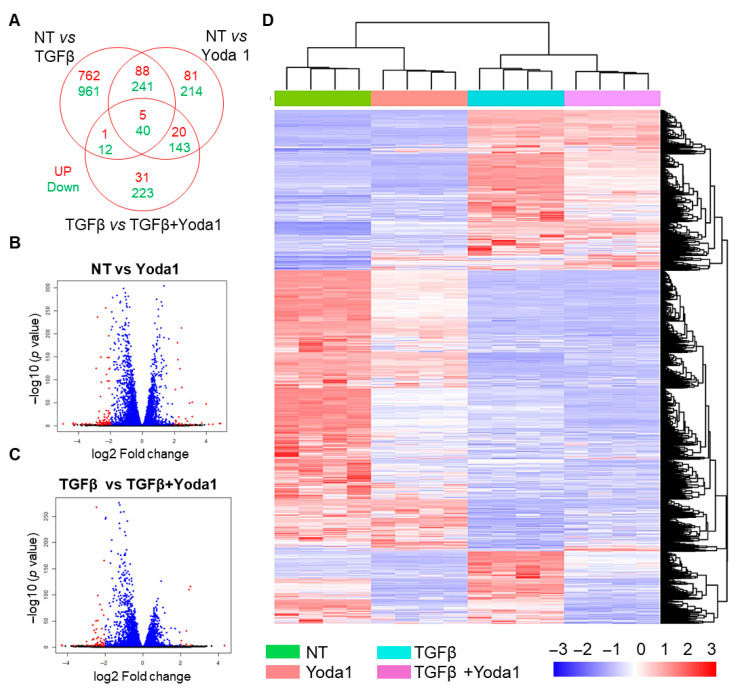
Gene expression profiling in NIH/3T3 fibroblasts: Effects of TGFβ and Yoda1. (**A**) The Venn diagram illustrates the overlap of upregulated and downregulated genes across the different study groups. Volcano plots comparing genes upregulated or downregulated in response to (**B**) Yoda1 versus control and (**C**) TGFβ versus TGFβ + Yoda1 in NIH/3T3 fibroblasts. The orange dots represent significantly upregulated or downregulated genes, whereas blue dots represent the genes with no significant difference in expression. (**D**) The heatmap displays the gene expression profiles of NIH/3T3 fibroblasts across the study groups: control, Yoda1, TGFβ, and TGFβ + Yoda1. NT = no treatment.

To investigate the molecular mechanism underlying Yoda1-induced inhibition of fibroblast activation, transcriptomic analysis was performed on TGFβ-treated NIH/3T3 cells in the presence and absence of Yoda1. Differential gene expression analysis revealed that 856 genes were upregulated and 1254 genes were downregulated in the TGFβ-treated group compared to the untreated control (≥2-fold change; *p* ≤ 0.05). In the Yoda1-treated group, 194 genes were upregulated and 638 genes were downregulated compared to the control (≥2-fold change; *p* ≤ 0.05) (Figure 4A,B). In the Yoda1 + TGFβ-treated group, 57 genes were upregulated and 418 genes were downregulated compared to TGFβ alone (≥2-fold change; *p* ≤ 0.05) (Figure 4A,C). Heatmap analysis further provided a global view of the differentially expressed genes across the four groups (control, TGFβ, Yoda1, and Yoda1 + TGFβ) (Figure 4D).

**Figure 5:**
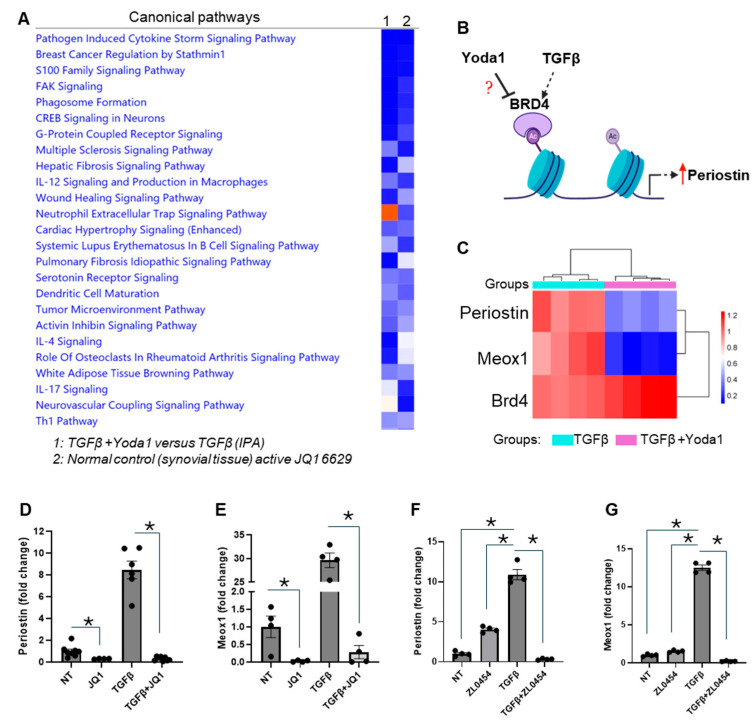
Inhibition of BRD4 had similar effects on NIH/3T3 fibroblast activation as Yoda1. (**A**) IPA was used to compare the top pathways affected in TGFβ + Yoda1 *versus* TGFβ (1) and *versus* normal control (synovial tissue) with JQ1 (a BRD4 inhibitor). (**B**) Graphical representation of the putative regulation of BRD4. Red arrows indicate upregulation, while question marks denote gaps in current knowledge. (**C**) The heatmap illustrates the relative expression levels of Periostin, Meox1, and BRD4 in TGFβ + Yoda1-treated cells compared to TGFβ alone. (**D**,**E**) Bar graphs showing the effects of JQ1 on the expression of Periostin and Meox1 with or without TGFβ treatment in NIH/3T3 fibroblasts. (**F**,**G**) Bar graphs showing the effects of ZL0454 on the expression of Periostin and Meox1 with or without TGFβ treatment in NIH/3T3 fibroblasts. Results are presented as mean ± SE. Statistical significance was determined using one-way ANOVA, with *p* value ≤ 0.05 considered statistically significant. * = *p* ≤ 0.05. NT = no treatment.

Additionally, we conducted a comparison analysis of differentially expressed genes between the Yoda1 + TGFβ and TGFβ-only groups using IPA. This comparison revealed that the pathways enriched in the Yoda1 + TGFβ group from our transcriptomic analysis closely resembled those affected by JQ1 treatment (Figure 5A). JQ1 is an inhibitor of the bromodomain and extra-terminal (BET) protein family, in particular BRD4, which appears to play a key role in mediating the inhibition of fibroblast activation induced by Yoda1 (Figure 5B). Although no significant difference was observed in the expression of BRD4, we detected a substantial reduction in the transcript levels of the fibroblast activation marker Periostin and Meox1, a transcription factor known to regulate pro-fibrotic gene expression (Figure 5C).

To explore the role of BRD4 in this process, NIH/3T3 fibroblasts were treated with the BRD4 inhibitors JQ1 and ZL0454, both with and without TGFβ. Our results demonstrated a significant reduction in Periostin expression following JQ1 treatment, both at baseline and after TGFβ treatment (Figure 5D). Additionally, JQ1 treatment inhibited the expression of Meox1in both the presence and absence of TGFβ (Figure 5E). Similarly, ZL0454 treatment reduced TGFβ-induced Periostin and Meox1 expression; however, no significant effect of ZL0454 was observed on baseline levels of Periostin or Meox1 (Figure 5F,G).

### 4.4. Overexpression of BRD4 Mitigates the Inhibitory Effect of Yoda1 on TGFβ-Induced Activation in Cardiac Fibroblasts

**Figure 6:**
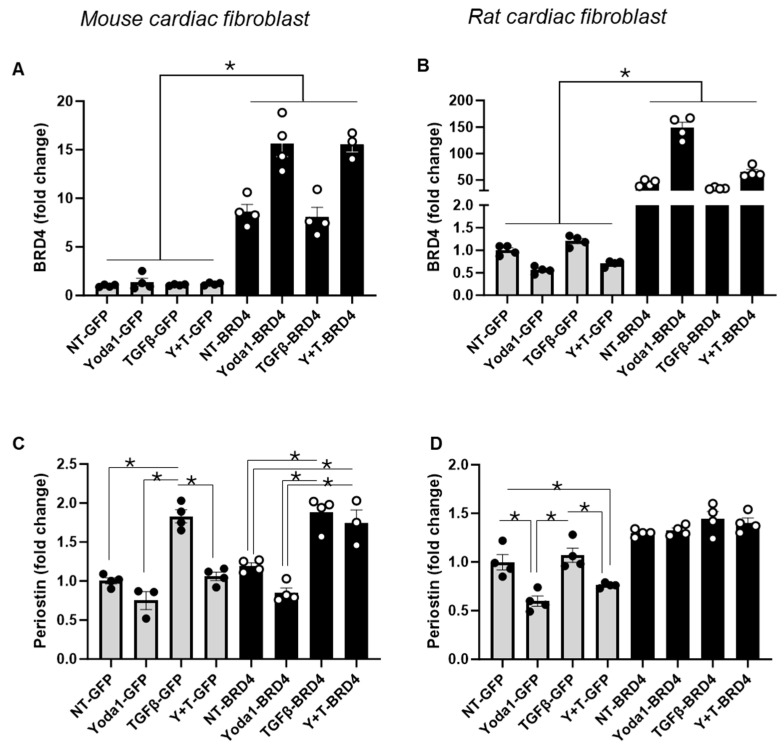
BRD4 overexpression mitigates the Yoda1-mediated inhibition of Periostin expression in cardiac fibroblasts. Bar graphs showing BRD4 expression in (**A**) mouse cardiac fibroblasts (CFs) and (**B**) rat CFs transduced with adeno-GFP or adeno-BRD4 and treated with TGFβ, Yoda1, or a combination. Bar graphs depicting Periostin expression in (**C**) mouse CFs and (**D**) rat CFs transduced with adeno-GFP or adeno-BRD4 and treated with TGFβ, Yoda1, or both. Results are presented as mean ± SE. Statistical significance was calculated using one-way ANOVA, with *p* ≤ 0.05 considered statistically significant. * = *p* ≤ 0.05. NT = no treatment; Y + T = Yoda1 + TGFβ.

To confirm that the inhibitory effect of Yoda1 on TGFβ-induced Periostin expression is mediated through BRD4 inhibition, we conducted BRD4 overexpression experiments using an in vitro model with mouse and rat CFs. Due to challenges with transfection and transduction in NIH/3T3 cells, data from these cells were not included. An adenovirus-based approach was employed to induce BRD4 overexpression. The ad-BRD4-mCherry virus was used to overexpress BRD4, while the ad-GFP virus served as a transduction control. After transduction, both the BRD4-overexpressing and GFP control groups were treated with Yoda1, TGFβ, or in combination. RT-PCR analysis confirmed successful overexpression of BRD4 in both mouse (Figure 6A) and rat cardiac fibroblasts (Figure 6B) following ad-BRD4-mCherry transduction versus control.

In mouse CFs, TGFβ treatment significantly induced Periostin expression in both the control (ad-GFP) and BRD4 overexpression groups. Notably, Yoda1 treatment inhibited TGFβ-induced Periostin expression in the control group, and this inhibition was reversed by BRD4 overexpression (Figure 6C). In rat CFs, TGFβ treatment did not significantly induce Periostin expression, but Yoda1 treatment still resulted in a significant reduction in Periostin expression, irrespective of TGFβ treatment. Similar to the mouse model, BRD4 overexpression in rat CFs blocked the inhibitory effect of Yoda1 on Periostin expression (Figure 6D). Overall, our results substantiate a BRD4-dependent mechanism in Yoda1-mediated inhibition of TGFβ-induced Periostin.

## 5. Discussion

Fibrosis is a pathological process that occurs in various organs and tissues under chronic injury conditions, contributing significantly to disease progression [39,40,41]. In the heart, fibrosis is particularly detrimental as it leads to excessive deposition of ECM components, disrupting normal myocardial architecture and impairing cardiac function [42,43]. CFs are the primary cell type responsible for ECM production, becoming activated in response to stress or injury. Upon activation, these fibroblasts proliferate and differentiate into myofibroblasts, a process essential for short-term tissue repair [44,45]. However, under chronic pathological conditions, persistent myofibroblast activity results in excessive ECM deposition, exacerbating fibrosis and further impairing heart function [46,47]. This dynamic process is largely governed by TGFβ, a potent cytokine that regulates fibroblast activation and myofibroblast differentiation [48,49].

Ion channels, in particular K^+^ channels, play a crucial role in regulating key cellular functions in fibroblasts, including maintaining ionic homeostasis, controlling membrane potential, and modulating calcium signaling, which are essential for fibroblast proliferation and activation [50,51,52,53]. However, the specific involvement of ion channels in TGFβ-induced cardiac fibroblast activation and the underlying mechanisms remain insufficiently explored. Therefore, this study investigates the role of ion channels in TGFβ-induced cardiac fibroblast activation, with a particular focus on K^+^ channels, mechanosensitive ion channels, and the potential molecular pathways involved.

Our findings demonstrate that TEA-sensitive channels modulate fibroblast activation, and Yoda1, a selective Piezo1 agonist, exerts inhibitory effects on TGFβ-induced fibroblast activation through a Piezo1-independent mechanism. Additionally, we identified BRD4 as a key mediator in this process. Our observation of reduced fibroblast activation after TEA treatment aligns with previous studies demonstrating the involvement of TEA-sensitive channels in cardiac fibrosis induced by reactive oxygen species or pressure overload [54,55,56]. Our data contribute to these findings by demonstrating that blockade of TEA-sensitive channels also inhibits fibroblast activation induced by TGFβ. However, we did not observe inhibitory effects from blocking BK, SK, or K_Ca_3.1 channels in TGFβ-induced fibroblast activation. While K_Ca_3.1 has been implicated in angiotensin II (AngII)-induced fibrosis [23,57], our results suggest that TGFβ-induced fibroblast activation operates through a distinct mechanism, independent of AngII signaling [58,59].

Our transcriptomic analysis revealed the activation of various critical fibrosis-associated pathways following TGFβ treatment. Furthermore, it highlights Piezo1 as one of the ion channels most highly expressed in 3T3 cells. Piezo channels are mechanosensitive ion channels that respond to mechanical forces such as stretch, and they play a role in cardiac fibrosis by regulating fibroblast activation, collagen deposition, and fibrosis progression [60,61,62,63]. Piezo1 in particular has been implicated in stress-induced fibrosis [60,61]. However, our investigation of its role in TGFβ-induced fibroblast activation yielded unexpected results. Pharmacological inhibition of Piezo1 did not affect TGFβ-induced fibroblast activation, yet Yoda1, a Piezo1 agonist, inhibited this process. Notably, the inhibitory effect of Yoda1 was not reversed by Piezo1 antagonists or siRNA-mediated knockdown of Piezo1. Our results showed no change in periostin expression across any treatment conditions following Piezo1 inhibition, compared to their respective controls without Piezo1 inhibition. While Piezo1 has been implicated in stress-induced fibroblast activation, we did not observe its significant role in TGF-βinduced cardiac fibroblast activation. This indicates that Piezo1 may contribute to fibroblast responses under mechanical stress but is unlikely to serve as a key mediator of TGFβ-driven fibroblast activation. Furthermore, the inability of Piezo1 antagonists or knockdown to reverse the inhibitory effect of Yoda1 points toward a Piezo1-independent mechanism underlying this response. Our investigation into the role of Piezo1 in TGFβ-induced cardiac fibroblast activation is the first to specifically examine this process, setting it apart from prior studies that have either focused on the role of Piezo1 in fibroblast maturation or used non-fibroblast cell types [64,65]. For example, Braidotti et al. demonstrated that cardiac fibroblasts exhibited a significant acquisition of the myofibroblast phenotype when cultured on stiff surfaces for 48 h compared to 24 h, but Piezo1 expression remained unchanged between these time points [64]. In contrast, Zhao et al. observed a stress-induced increase in Piezo1 expression in human HK2 cells after five days of culture on polyacrylamide hydrogel-coated plates with modified surface stiffness [65]. This study further revealed Piezo1-mediated activation of pro-fibrotic genes following TGFβ treatment, an effect that was inhibited by the Piezo1 inhibitor GsMTx4 at 48 h post-treatment [65].

Taken together, these studies suggest that Piezo1 may be more prominent in the later stages of fibroblast differentiation rather than in early activation. This hypothesis is reinforced by the timing of data collection in these studies, which examined responses at later time points (48 h or 5 days), whereas our study assessed responses at 24 h. Additionally, the role of Piezo1 in fibrosis appears to be both contextual and cell-type-dependent, as Zhao et al. used HK2 cells, which are not of fibroblast origin. Our findings support the notion that Piezo1 may have distinct and context-dependent roles in fibrosis, particularly when comparing mechanical, stress-induced fibroblast activation versus TGFβ-induced fibroblast activation. However, to fully delineate Piezo1’s specific contributions to fibrosis, additional mechanistic studies are needed, particularly those that focus on different fibrotic contexts and cell types. These future studies will be crucial in understanding the complex role of Piezo1 in fibrosis and its potential as a therapeutic target.

Our RNA-seq analysis also revealed a significant overlap in gene expression profiles between Yoda1- and JQ1-treated fibroblasts. JQ1 is a known BRD4 inhibitor [66]. BRD4, a member of the BET (bromodomain and extra-terminal) protein family, modulates gene expression by interacting with acetylated histones and other proteins, influencing chromatin structure and transcription [67,68]. BRD4 has been implicated in cardiac fibrosis [69], but its role in TGFβ-induced fibroblast activation and its interaction with Yoda1 remain unexplored. Our results demonstrate that BRD4 inhibition, using either JQ1 or ZL0454, significantly reduced the expression of fibrosis-related genes such as Periostin and Meox1, which are critical regulators of fibroblast activation and ECM deposition [45,69]. Importantly, overexpression of BRD4 reversed the inhibitory effects of Yoda1, which substantiates its role in TGFβ-induced fibroblast activation. These findings position BRD4 as a key mediator in Yoda1-induced inhibition of TGFβ-driven fibroblast activation, providing new insights into potential therapeutic strategies targeting BRD4 in fibrosis.

While our study provides valuable insights into the molecular mechanisms underlying TGFβ-induced fibroblast activation, it also raises several important questions and underscores the need for further investigation to fully elucidate the mechanisms driving fibroblast activation under diverse conditions and stimuli. First, although the BRD4-mediated effect of Yoda1 on fibroblast activation represents a novel finding, the precise molecular interactions between Yoda1, Piezo1, and BRD4 that contribute to the inhibition of fibroblast activation remain unclear and require detailed exploration. Given that Yoda1 has no effect on BRD4 expression levels, it appears likely that Yoda1 may interfere with BRD4 binding, similar to JQ1. Additionally, our findings suggest that stress-induced and TGFβ-induced fibroblast activation may operate through distinct mechanisms, a hypothesis that merits in-depth investigation to clarify the context-dependent roles of these pathways. Beyond mechanistic insights, the clinical potential of targeting BRD4 in fibrotic diseases, including cardiac fibrosis, presents a promising avenue for future research. Our findings highlight the therapeutic potential of modulating these pathways, offering novel strategies for the prevention and treatment of fibrosis in the heart and other organs.

In conclusion, this study provides critical insights into the molecular mechanisms underlying TGFβ-induced cardiac fibroblast activation, highlighting the roles of TEA-sensitive ion channels and BRD4-dependent pathways in regulating this process. Our results also reveal the novel role of Yoda1 in inhibiting TGFβ-induced fibroblast activation and underscore mechanistic differences between stress-induced and TGFβ-induced fibrosis.

## Figures and Tables

**Table 1:** Details of chemicals used, including concentrations and suppliers.

Name	Concentration	Company/Catalog Number
TGFβ	10 ng/mL	Gibco™ (Waltham, MA, USA)/PHG9204
Yoda1	10 µM	Sigma-Aldrich (St. Louis, MO, USA)/SML1558-5MG
TEA	10 mM	Sigma-Aldrich (St. Louis, MO, USA)/8083520100
4-AP	2 mM	Sigma-Aldrich (St. Louis, MO, USA)/A78403-25G
Ibtx	100 nM	Tocris Bioscience™ (Minneapolis, MN, USA)/10-86100U
Apamin	100 nM	Sigma-Aldrich (St. Louis, MO, USA)/A1289-1mg
TRAM34	100 nM	Sigma-Aldrich (St. Louis, MO, USA)/T6700-25mg
BaCl_2_	100 µM	Sigma-Aldrich (St. Louis, MO, USA)/B-0750
GsMTx4	2.5 µM	Tocris Bioscience™ (Minneapolis, MN, USA)/49-121-00U
JQ1	10 µM	Sigma-Aldrich (St. Louis, MO, USA)/SML1524-5MG
ZL0454	10 µM	Medchemexpress LLC (Monmouth Junction, NJ, USA) /HY1121505MG

**Table 2:** List of primers.

Gene Name	Species	Orientation	5′-3′ Sequence
Periostin	Mouse	Forward	CCTGCCCTTATATGCTCTGCT
	Mouse	Reverse	AAACATGGTCAATAGGCATCACT
Meox1	Mouse	Forward	GAAACCCCCACTCAGAAGATAGC
	Mouse	Reverse	TCGTTGAAGATTCGCTCAGTC
UBC	Mouse	Forward	CCCAGTGTTACCACCAAGAAG
	Mouse	Reverse	CCCCATCACACCCAAGAACA
BRD4	Mouse	Forward	CTGCTCAGAGTGGTGCTCAA
	Mouse	Reverse	TGCTGCCCTACCTGTTTCTT
αSMA	Mouse	Forward	GCTTCGCTGGTGATGATGCTC
	Mouse	Reverse	AGTTGGTGATGATGCCGTGTTC
Periostin	Rat	Forward	TGCAAAAAGACACACCTGCAA
	Rat	Reverse	CCGAAGTCAATGGGGCTCTT
Meox1	Rat	Forward	AGTTCGCCCACCATAACTACC
	Rat	Reverse	TTGGAAAGGGCGTTCGTTCA
UBC	Rat	Forward	ACACCAAGAAGGTCAAACAGGA
	Rat	Reverse	GACACCTCCCCATCAAACCC

**Table 3:** Expression profile of ion channels in NIH/3T3 fibroblasts: A focused analysis of ion channel expression identified Piezo1 as the most highly expressed ion channel. Ion channels are listed in descending order of expression (highest at the top). For each channel, abbreviations, channel type, and full names have been provided.

	Gene	Channel Type	Channel Name
1	*Piezo1*	Cation	Piezo-type mechanosensitive ion channel component 1
2	*Vdac1*	Anion	Voltage-dependent anion channel 1
3	*Pkd2*	Cation	Polycystin 2, transient receptor potential cation channel
4	*Trpm7*	Cation	Transient receptor potential cation channel, subfamily M, member 7
5	*Vdac2*	Anion	Voltage-dependent anion channel 2
6	*Vdac3*	Anion	Voltage-dependent anion channel 3
7	*Clns1a*	Chloride	Chloride channel, nucleotide-sensitive, 1A
8	*Cacna1c*	Calcium	Calcium channel, voltage-dependent, L type, alpha 1C subunit
9	*Clcn5*	Chloride	Chloride channel, voltage-sensitive 5
10	*Clcn4*	Chloride	Chloride channel, voltage-sensitive 4
11	*Cacna1g*	Calcium	Calcium channel, voltage-dependent, T type, alpha 1G subunit
12	*Cacng7*	Calcium	Calcium channel, voltage-dependent, gamma subunit 7

## Data Availability

The data supporting the findings of this study are available from the corresponding author upon reasonable request.
